# From prioritization to implementation: updating the PARC WP5 project portfolio through the second prioritization round

**DOI:** 10.3389/ftox.2026.1746437

**Published:** 2026-02-09

**Authors:** Celia Garcia Arenas, Kiara Aiello Holden, Terje Svingen, Dries Knapen, Saadia Kerdine-Römer, Birgitte Lindeman, Nicola M. Smith, Ludovic Le Hegarat, Tamara Vanhaecke, Gilles Rivière, Philip Marx-Stoelting

**Affiliations:** 1 Department of Pesticides Safety, German Federal Institute for Risk Assessment, Berlin, Germany; 2 National Food Institute, Technical University of Denmark, Kgs Lyngby, Denmark; 3 Department of Veterinary Sciences, University of Antwerp, Wilrijk, Belgium; 4 Inflammation microbiome immunosurveillance, Inserm, Université Paris-Saclay, Orsay, France; 5 Department of Chemical Toxicology, Norwegian Institute of Public Health, Oslo, Norway; 6 French Agency for Food Environmental and Occupational Health and Safety, Maisons-Alfort, France; 7 Department of In Vitro Toxicology and Dermato-Cosmetology, Vrije Universiteit Brussel, Jette, Belgium

**Keywords:** chemical hazard assessment, NAMs, new approach methods, next-generation risk assessment, partnership for the assessment of risks from chemicals, regulatory readiness

## Abstract

The Partnership for the Assessment of Risks from Chemicals (PARC) represents a joint effort among risk assessors, regulatory authorities, and the scientific community to advance the implementation of next-generation risk assessment (NGRA) in line with the objectives of the EU Chemicals Strategy for Sustainability. Addressing the challenges faced by the national and European regulators by integrating data generated through innovative methodologies is central to achieving this goal. Following an initial phase of the partnership, in which projects were defined based on a first prioritization of methodologies, a second prioritization round was conducted with input from the Governing Board representatives of all participating entities. This second process also considered the Key Areas of Regulatory Challenge introduced by the European Chemicals Agency in 2023, ensuring that the evolving research agenda within PARC is closely aligned with current and future regulatory needs. As a result, WP5 Hazard Assessment has updated its project portfolio to include four new projects that bridge identified regulatory gaps and strengthen the implementation phase of PARC. One project focuses on developmental and reproductive toxicity, another on developmental immunotoxicity, a third explores sexual dimorphism associated with hepatotoxicity, and a fourth transversal project, Regulatory Readiness of NAMs, aims to accelerate the regulatory uptake of promising methods developed under Work Package 5 (WP5). This article complements the previous PARC special issue by providing an overview of the updated WP5 project portfolio, illustrating the progression from prioritization to implementation, and highlighting how these new projects respond to evolving regulatory needs and contribute to the effective integration of new approach methodologies (NAMs) into chemical risk assessment.

## 1 Introduction

The Partnership for the Assessment of Risks from Chemicals (PARC) was launched in May 2022 to advance innovative approaches for chemical risk assessment across Europe. PARC was designed to address many of the persistent challenges in the development and implementation of new approach methodologies (NAMs) in modern chemical risk assessment, bringing together academia, national agencies, and EU regulatory bodies to accelerate the shift toward predictive, mechanistic, and human-relevant safety assessments (Marx-Stoelting et al., 2023).

As outlined in our previous review published in this PARC special issue (De Castelbajac et al., 2023), Work Package 5 (WP5) (hazard assessment) focuses on developing NAMs that help fill data gaps (especially key events in adverse outcome pathways (AOPs)), enable grouping and read-across approaches, and inform integrated approaches to testing and assessment (IATAs).

PARC projects and activities provide direct added value to regulatory risk assessment and management at both the EU and national levels. The identification of the first set of priorities for PARC began before the launch of the partnership, building on the legacy of HBM4EU, a Horizon 2020 project that aimed to coordinate and advance human biomonitoring in Europe (https://cordis.europa.eu/project/id/733032), and a survey distributed to interim Governing Board (GB) members (Rousselle and Tannous, 2023).

The first round of prioritization of methods for WP5 followed criteria that considered the survey results, the objectives of the EU Chemicals Strategy for Sustainability (CSS), and the feasibility and expertise of the partners. As a result, five endpoints were prioritized, and the following five corresponding projects were launched:

- Non-genotoxic carcinogenicity (NGTxC) (Audebert et al., 2023).
- Metabolic endocrine disruption (MD-ED) (Braeuning et al., 2023).
- Endocrine disruption (thyroid) (ED-Th) (Ramhøj et al., 2023).
- Immunotoxicity (Immunotox) (Snapkow et al., 2024).
- (Developmental and Adult) Neurotoxicity (DNT-ANT) (Tal et al., 2024).

A second prioritization round, which was initiated in 2023, aimed to map the needs for the second half of PARC (2025–2029). Feedback from PARC experts and GB principal representatives in the first round was collected to ensure a transparent process, maintain a balance between short-term regulatory needs and long-term innovation, avoid duplication with other EU initiatives, and foster collaboration across PARC tasks and work packages. Each GB representative could suggest new substances or substance groups, endpoints, methods, and case studies for inclusion in PARC’s future priorities list.

In parallel, in 2023, the European Chemical Agency introduced the Key Areas of Regulatory Challenge (KARCs):

- Protection against the most harmful chemicals.
- Addressing chemical pollution in the environment.
- Shifting away from animal testing.
- Improving the availability of chemical data.

The results of the second prioritization round showed that this mapping exercise was highly valuable, although it produced an extensive “wish list.” Alignment between the survey results and the KARC priorities is, therefore, needed to maximize regulatory impact while balancing ongoing work and partner capacity (Tannous and Rousselle, 2024).

Within WP5 Hazard Assessment, four new projects were launched in 2025 to address newly identified regulatory gaps:

- Developmental and reproductive toxicity (DART).
- Developmental immunotoxicity (DIT).
- Sexual dimorphism associated with hepatotoxicity (SD-Hepatox).
- Regulatory readiness of NAMs (RegNAMs).

The first three projects focus on specific toxicity endpoints, while RegNAMs is a transversal project aimed at accelerating the regulatory uptake of NAMs. This project supports pre-validation activities and systematic evaluation of NAMs with high potential regulatory impact. These coordinated efforts aim to fill critical data gaps, harmonize with evolving regulatory frameworks, and accelerate the implementation of next-generation risk assessment (NGRA) in chemical safety (Marx-Stoelting et al., 2024).

**FIGURE 1:**
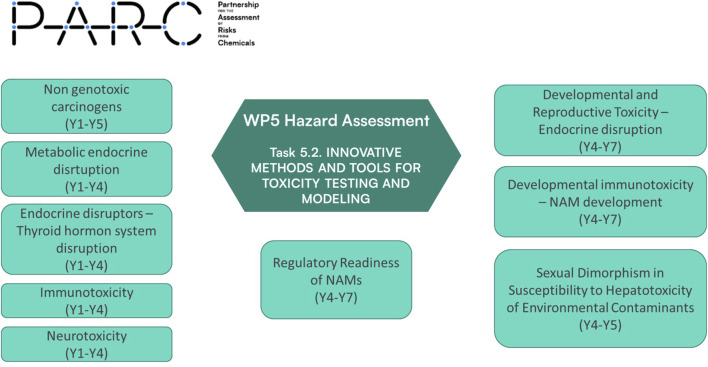
Work Package 5 organigram, focusing on projects under Task 5.2 “Innovative tools and methods for toxicity testing (NAMs) for human health.”

This article is a follow-up to our previous review and presents an updated overview of the PARC WP5 portfolio (Figure 1), integrating outcomes from the second prioritization round and alignment with KARC priorities.

## 2 Developmental and reproductive toxicity

### 2.1 Context

Developmental and reproductive toxicity (DART) mediated by endocrine disruption (ED) is of critical concern for human and environmental health, yet integrated methods to assess DART–ED interactions are still limited, particularly in non-mammalian systems. Current OECD test guidelines and REACH requirements do not fully capture the mechanistic links between endocrine activity and developmental or reproductive outcomes (Holmer et al., 2025; Knapen et al., 2025). Advancing integrated DART–ED approaches in fish and *in vitro* models will fill major regulatory gaps, enable cross-species extrapolation, and support the broader adoption of mechanistically based, non-animal methods in chemical risk assessment.

### 2.2 How?

This project will provide mechanistic evidence to support chemical safety assessors and regulators by facilitating the increased use of non-animal test data for DART–ED identification and decision-making. Specifically, the project will

- Deliver ED-sensitive endpoints for addition to fish test guidelines for both non-protected (TG 236) and protected life stages (e.g., TG 210, required under Annex IX of REACH).
- Develop ED endpoints for placental toxicity, which is relevant for both reproductive and developmental assessment.
- Advance *in vitro* models for male steroidogenesis and testis function.

### 2.3 Innovation and regulatory impact

This project addresses regulatory needs over multiple time frames. In the short term, work on DART–ED methods in fish will build on and complement the ongoing validation of THSD-sensitive endpoints for inclusion in existing OECD test guidelines (TG 236, 210) under the validation management group for ecotoxicology. Integrating ED-sensitive endpoints into these guidelines and REACH requirements will fill the current gap in environmental ED evaluation.

In the medium term, the development of a NAM to assess placental angiogenesis and endocrine disruption will support the characterization and classification of reproductive EDs and may contribute to the weight of evidence for AOP 151 (OECD Project 1.42, under development).

In the long term, replacing the H295R *in vitro* steroidogenesis assay with more relevant alternatives is crucial for NAM-based assessments of male reproductive toxicants. The project will deliver key outcomes for the development of such models, thus contributing to validated *in vitro* NAMs for male steroidogenesis.

## 3 Developmental immunotoxicity

### 3.1 Context

Developmental immunotoxicity (DIT) is a growing concern because the developing immune system is highly sensitive, and disruption of the immune system can lead to long-term health effects, such as allergies, autoimmune diseases, and increased susceptibility to infections (Hessel et al., 2015). Current chemical regulations offer limited tools to identify immunotoxicants as functional immune endpoints are underrepresented in standard test guidelines. The T-cell-dependent antibody response (TDAR) in the Extended One-Generation Reproductive Toxicity Study (EOGRTS) is the only functional assay required, and the DIT cohort is rarely included due to the absence of validated triggers. Recognizing these limitations, ECHA has identified DIT as a key regulatory challenge, calling for the identification of critical developmental windows and the development of NAMs. Recent OECD activities and guidance (e.g., DRP No. 360 (OECD, 2022)) and frameworks such as the “Key Characteristics of Immunotoxic Agents” (Germolec et al., 2022) provide a solid foundation for advancing non-animal methods and building a NAM-based DIT testing strategy.

### 3.2 How?

In line with ECHA’s KARC recommendations for DIT, this project will

- Identify critical developmental windows (through physiological mapping and literature review) to support NAM development.
- Provide screening and prioritization tools using existing assays and NAMs.
- Generate mode-of-action information to support read-across for DIT using existing assays and NAMs.
- Evaluate existing assays as potential NAMs to support triggering decisions for the inclusion of DIT cohorts in EOGRTS.
- Develop new NAMs specifically targeting developmental processes to lay the foundation for a test battery for DIT compound identification.

### 3.3 Innovation and regulatory impact

The DIT project is directly relevant to multiple regulatory frameworks—including REACH, CLP, the Biocidal Products Regulation, the Plant Protection Products Regulation, and the Cosmetics Products Regulation (OECD, 2025; Dewhurst et al., 2015]—by providing tools for screening, prioritization, read-across, and grouping proposals. Several existing immunotoxicity assays are well-characterized, and some have been developed into OECD test guidelines (e.g., TG 444 on skin sensitization) (OECD, 2023). These constitute a valuable source of methods to be evaluated for their potential to serve as NAMs addressing specific regulatory needs. Further adaptation of these assays to include cell maturation and migration endpoints will be explored. In the long term, new models based on hematopoietic stem cells and hiPSCs will contribute to the development of non-animal tools for hazard identification and characterization, thereby enhancing the overall assessment of DIT properties.

## 4 Sexual dimorphism associated with susceptibility to hepatotoxicity of environmental contaminants

### 4.1 Context

Given its central role in xenobiotic metabolism, the liver represents a primary target organ for chemical-induced toxicity (Rusyn et al., 2021). Increasing evidence indicates that chemical exposures may cause differential effects in men and women, primarily due to sex-specific variations in hepatic metabolic pathways (Hochmuth et al., 2021; Zuri, 2024). Notably, women account for approximately 70% of acute liver-failure cases, indicating a greater susceptibility to hepatotoxicants (Sayaf et al., 2022). While animal studies may partially address sexual dimorphism by selecting the more sensitive sex, current *in vitro* liver models generally fail to capture these biological differences, thereby limiting their predictive capacity for sex-specific liver toxicity responses.

### 4.2 How?

This project aims to improve *in vitro* models for chemical-induced hepatotoxicity by incorporating parameters of sexual dimorphism into hepatic cell systems, including primary human hepatocytes, HepaRG cells, and hepatocytes derived from multipotent stem cells (mSCs) and induced pluripotent stem cells (iPSCs). Specifically, the work will carry out the following steps:

- Establish and characterize sexual dimorphism in *in vitro* hepatic models.
- Assess the effects of prototypical compounds with known sex-specific impacts on liver functionality using the established models.
- Evaluate sex-specific hepatotoxicity responses of the hepatic models to selected environmental contaminants from the PARC priority list.

### 4.3 Innovation and regulatory impact

From a regulatory perspective, this project will, for the first time, enable the integration of sexual dimorphism and sex-specific responses in chemical hazard analysis, ultimately supporting better protection of public health and the environment.

This project represents a proof-of-concept (PoC) effort to evaluate the biological relevance of sexual dimorphism in an *in vitro* context, including assessments of chemical-specific responses (e.g., endocrine disruptors). The approach will enhance mechanistic understanding, which is a prerequisite for developing or refining *in vitro* test systems and NAMs for defined regulatory contexts. Should PoC data demonstrate robust biological differences, all NAMs currently—or prospectively—used in regulatory frameworks could be affected. Further qualification and validation of these assays will be needed before formal adoption; however, even without full validation, such NAMs could contribute to weight-of-evidence assessments alongside animal studies, *in silico* models, and historical data.

## 5 Regulatory readiness of NAMs

### 5.1 Context

Current regulatory frameworks for chemical safety rely predominantly on OECD test guidelines, yet many promising NAMs remain underutilized because their regulatory readiness is insufficient (Schmeisser et al., 2023). Within PARC, several NAMs have been developed or refined under WP5, but further work is needed to align these approaches with regulatory expectations (Marx-Stoelting et al., 2024). Enhancing regulatory readiness is crucial to ensuring that NAMs contribute effectively to hazard assessment, screening, and decision-making, thereby supporting the 3Rs principle and improving the protection of human health and the environment.

This project specifically addresses the need to evaluate, harmonize, and advance the most promising NAMs toward regulatory implementation.

### 5.2 How?

The project will support pre-validation and readiness activities for selected NAMs by

- Expanding the ReadEDTest framework to assess methods for priority endpoints within WP5.
- Organizing independent peer reviews of NAM readiness, including SOPs and ReadEDTest outcomes.
- Selecting NAMs with the highest regulatory relevance for further development
- Supporting (pre-)validation activities—including SOP refinement (e.g., through good *in vitro* method practices workshops), alignment with AOPs and IATAs, and coordination with the European Union Network of Laboratories for the Validation of Alternative Methods (EU-NETVAL), WP6—Innovation in Regulatory Risk Assessment, and other relevant initiatives—this project will promote coherence across validation efforts. To build regulatory confidence, selected NAMs will be applied in parallel with conventional test guideline studies where feasible, transferred across laboratories to assess reproducibility, and integrated into AOP- and IATA-based test batteries for complex endpoints.

### 5.3 Innovation and regulatory impact

Rather than pursuing full OECD-style validation, this project emphasizes structured evidence generation to support regulatory confidence, including the parallel use of NAMs with established methods, assessment of transferability, and integration of promising assays into AOP- and IATA-based test batteries for complex endpoints.

By systematically improving the regulatory readiness of selected NAMs, this project will facilitate their integration into chemical safety assessments. The results will support hazard characterization, prioritization, and weight-of-evidence evaluations across multiple frameworks, including REACH, CLP, the Biocidal Products Regulation, the Plant Protection Products Regulation, and the Cosmetics Regulation.

Strengthening regulatory applicability will ensure that NAMs contribute to science-based decision-making and the progressive reduction of animal testing, thereby producing tangible benefits for human and environmental protection.

## 6 Concluding remarks

This manuscript summarizes the evolution of the PARC WP5 portfolio following the second prioritization round, highlighting the newly launched projects that address key regulatory needs identified through the KARC framework. Collectively, these initiatives represent an important step from prioritization to implementation, thereby advancing integrated, mechanistic, and non-animal approaches for chemical hazard assessment.

The addition of projects on DART, DIT, sexual dimorphism in hepatotoxicity, and regulatory readiness of NAMs strengthens PARC’s capacity to deliver fit-for-purpose tools with tangible regulatory impact, in line with the objectives of the EU Chemicals Strategy for Sustainability. As the partnership progresses, the harmonization of methods, validation strategies, and cross-work-package collaboration will be essential to ensure that scientific innovation is effectively translated into regulatory practice.

In the future, new and follow-up projects in 2026 will build on this strategy by enhancing the readiness and regulatory applicability of WP5 methodologies, thus supporting continued progress toward the uptake of NAMs and the long-term sustainability of PARC outcomes.
